# Bacteria viability assessment after photocatalytic treatment

**DOI:** 10.1007/s13205-013-0137-1

**Published:** 2013-05-21

**Authors:** Yanling Cai, Maria Strømme, Ken Welch

**Affiliations:** Division for Nanotechnology and Functional Materials, Department of Engineering Sciences, The Ångström Laboratory, Uppsala University, Box 534, 75121 Uppsala, Sweden

**Keywords:** Photocatalysis, Bacterial viability, Live/Dead staining, Metabolic activity assays, CFU counting

## Abstract

The aim of the present work was to evaluate several methods for analyzing the viability of bacteria after antibacterial photocatalytic treatment. Colony-forming unit (CFU) counting, metabolic activity assays based on resazurin and phenol red and the Live/Dead^®^ *Bac*Light™ bacterial viability assay (Live/Dead staining) were employed to assess photocatalytically treated *Staphylococcus epidermidis* and *Streptococcus mutans*. The results showed conformity between CFU counting and the metabolic activity assays, while Live/Dead staining showed a significantly higher viability post-treatment. This indicates that the Live/Dead staining test may not be suitable for assessing bacterial viability after photocatalytic treatment and that, in general, care should be taken when selecting a method for determining the viability of bacteria subjected to photocatalysis. The present findings are expected to become valuable for the development and evaluation of photocatalytically based disinfection applications.

## Introduction

Photocatalysis of titanium dioxide (TiO_2_) has been widely investigated and successfully applied in a wide variety of applications such as solar cells (Nah et al. 2010), disinfection, anti-fouling and self-cleaning surfaces (Chen and Poon 2009; Robertson et al. 2012; Sanchez et al. 2012). When the anatase crystalline form of TiO_2_ is irradiated with light having a wavelength less than 385 nm, an electron–hole pair is generated as electrons are excited above the material’s band gap of 3.2 eV. TiO_2_ can also be doped to change the band gap energy and thereby enable the photocatalytic process under visible light (Chatterjee and Dasgupta 2005; Jie et al. 2012; Sheng et al. 2009). The excited electrons can react with oxygen to produce a superoxide ion (O_2_^·−^), while the positive holes can react with H_2_O or OH^−^ to produce hydroxyl radicals (·OH). Further reactions can generate other reactive oxygen species (ROS) like hydroxyl peroxide (H_2_O_2_) and singlet oxygen (^1^O_2_) (Chen and Poon 2009; Fujishima et al. 2008). The ROS generated by TiO_2_ photocatalysis have been proved to provide an antibacterial effect by many researchers (Welch et al. 2010; Li et al. 2008; Sanchez et al. 2012; Robertson et al. 2012). This disinfection ability of photocatalytic materials is due to the high redox reaction ability of the photocatalytic products, and the primary mechanism is thought to be the destruction of the cell membrane or cell wall causing leakage or structural damage of the cell (Maness et al. 1999). Research has demonstrated killing of viruses, Gram-positive and Gram-negative bacteria and even cancer cells by the photocatalysis of TiO_2_ (Li et al. 2008; Blake et al. 1999; Welch et al. 2010).

There is a growing interest in applying TiO_2_ photocatalysis to disinfection and antibacterial applications (Allahverdiyev et al. 2011; Robertson et al. 2012; Sanchez et al. 2012; Welch et al. 2010; Lilja et al. 2012). To produce reliable results from research, it is critical to have accurate and high-throughput methods for screening bacterial viability after photocatalytic treatment. When assessing bacterial viability and, in particular, bacteria in biofilm form, it is often necessary to use several methods in concert to get reliable results. Currently, methods widely used in bacterial viability analysis include indirect methods based on further culture of bacterial samples or direct methods based on molecular probes.

Colony-forming unit (CFU) counting is a conventional indirect method for assessing viability based on cell counting. Given the assumption that each viable bacterium grows and forms a colony, CFU counting method provides advantages like sensitivity (very low concentrations of living bacteria can be determined) and only counts viable cells. However, CFU counting is not a reliable method for bacteria forming clumps or chains, and especially biofilms, which are the prevalent growth form of most bacteria found in nature (Bettencourt et al. 2010). Furthermore, CFU counting after serial dilution and plating is a labor-intensive and time-consuming process, which hinders its application in high-throughput experiments.

A group of indirect methods for quantifying live bacteria, and even biofilms, is based on the detection of metabolic activity. A number of different indicators are used for this purpose, including resazurin (Sandberg et al. 2009), fluorescein diacetate (FDA) (Diaper et al. 1992), tetrazolium salt (XTT) (Belanger et al. 2011) and pH indicators like phenol red (Pantanella et al. 2008). These metabolic activity tests rely on the production of detectable signals resulting from a reaction between the indicator and the metabolite intermediate (e.g., NADPH) or product (e.g., lactic acid) (Peeters et al. 2008). The intensities of the detectable signals are assumed to be proportional to the viability of the bacterial samples, which depend on both the number of bacteria and metabolic rate of the bacteria. An advantage of metabolic activity detection is the ability to avoid or minimize sample manipulation, which makes these methods more suitable for high-throughput screening (Belanger et al. 2011). A limitation of metabolic activity detection is the uncertainty arising from the variation in innate metabolic rates of different bacteria. For example, different strains of the same bacterial species or the same bacteria strain in planktonic or biofilm form may have different growth rates (Welch et al. 2012; Mah and O’Toole 2001; Donlan 2001).

Molecular probe assays are direct methods for bacterial viability detection that do not require further culturing. Cell membrane integrity is typically considered a criterion of cell viability and is, thus, used in molecular probe assays. There are many commercially available kits based on fluorescent dyes, such as the Live/Dead^®^*Bac*Light™ bacterial viability assay kit (Live/Dead staining) containing SYTO 9 and propidium iodide dyes (Berney et al. 2007; Bar et al. 2009), the redox activity assay based on CTC or RedoxSensor™ Green reagent (Asadishad et al. 2011) and the *Bac*Light™ Bacterial Membrane Potential Kit based on DiOC_2_(3) (Lisle et al. 1999). The Live/Dead staining is a widely used method and utilizes both SYTO 9, which has a green fluorescence emission and stains both live and dead bacterial DNA, and propidium iodide, which has a red fluorescence emission and penetrates only damaged cell membranes. When the fluorescence is measured directly (e.g., with a microplate reader) or combined with flow cytometery, bacterial viability can be detected rapidly and accurately (Berney et al. 2007), while when assessed with fluorescent microscopy or laser scanning confocal microscopy (LSCM), regions of varying viability can be differentiated with imagery (Wierzchos et al. 2004).

In this work, we evaluated and compared several methods for analyzing bacteria treated with TiO_2_ photocatalysis, including CFU counting, metabolic activity assays based on resazurin and phenol red and Live/Dead staining. The methods were applied on two different bacterial strains: *Staphylococcus* *epidermidis* and *Streptococcus* *mutans*. *S.* *epidermidis* was chosen because it is a common cause of infections on skin-penetrating implants (Collinge et al. 1994; Mahan et al. 1991), and such infections are of interest to prevent using photocatalysis (Lilja et al. 2012). *S.* *mutans* was chosen because it is one of the initial colonizers in the formation of dental plaque and plays an important role in acid production leading to the development of dental cavity (Banas 2004). Applications of photocatalysis in dental materials could be used, for example, to reduce secondary dental caries following dental restoration (Welch et al. 2010).

## Materials and methods

### Bacterial strains and culture medium

Two bacterial strains, *S.* *epidermidis* (CCUG 18000A) and *S.* *mutans* (NCTC 10449), were employed to evaluate the conformity of the different viability quantification methods after TiO_2_ photocatalytic treatment. *S.* *epidermidis* was employed in planktonic form, while *S.* *mutans* was employed in both planktonic and biofilm form, depending on the quantification method. *S.* *epidermidis* was inoculated in 20 mL cation-adjusted Mueller–Hinton (MH) Broth (Fluka,Sigma-Aldrich Chemie GmbH, Steinheim, Germany) and cultured at 37 °C under agitation to late log phase. *S.* *mutans* was inoculated into Brain–Heart Infusion (BHI) broth (Fluka, Sigma-Aldrich Chemie GmbH, Steinheim, Germany) culture medium and cultured overnight at 37 °C.

Before the photocatalytic treatment, bacteria were collected by centrifugation (4,000 rpm, 10 min, EBA 30 centrifuge, Hettich, Tuttlingen, Germany) and re-suspended in sterile deionized water to achieve the desired concentration of bacteria for the tests involving planktonic bacteria. Bacterial concentration was determined by optical density measurements. The *S.* *mutans* biofilm preparation procedure is described below in the section for LSCM.

### Photocatalytic test surfaces

In this study, resin-based nanocomposite disks comprising a dental adhesive containing TiO_2_ nanoparticles, hereafter referred to as NP adhesives, were used as a standard photocatalytic surface. The photocatalytic nanoparticles used in this work were P25 TiO_2_ nanoparticles (lot number 4166031598, Evonik Industries (previously Degussa) AG, Germany), which consist of anatase and rutile crystalline phases of TiO_2_ in a ratio of about 3:1. The average sizes of anatase and rutile elementary particles are 25 nm and 85 nm, respectively (Ohno et al. 2001; Kirchnerova et al. 2005). NP adhesives have been proved to possess sufficient photocatalytic activity for achieving bacteria (Welch et al. 2010) and even biofilm elimination (Cai et al. 2013).

The light-cured dental adhesive resin was made by mixing 2, 2-bis [4-(2-hydroxy-3- methacryloxypropoxy) phenyl]-propane (BisGMA, Polysciences Europe GmbH, Eppelheim, Germany) and 2-hydroxyethyl methacrylate (HEMA, Sigma-Aldrich, Schnelldorf, Germany) in a 55/45 wt/wt ratio. Photoinitiator and coinitiators were added as follows: 0.5 mol % camphorquinone (CQ); 0.5 mol % 2-(dimethylamino) ethyl methacrylate (DMAEMA); 0.5 mol % ethyl-4-(dimethylamino) benzoate (EDMAB); and 1 wt % diphenyliodonium hexafluorophosphate (DPIHP) (all from Sigma-Aldrich, Steinheim, Germany).

The NP adhesive disks were made by mixing 20 wt % P25 TiO_2_ nanoparticles with the adhesive resin. The disks were cast in circular Teflon molds (diameter 8 mm, thickness 1 mm) and light-cured with 460 nm light for 30–40 s (BlueLEX GT1200, Monitex, Taiwan) under N_2_ flow. Sample disks were randomly grouped for the different viability test methods.

### Antibacterial treatments

Prior to antibacterial treatment, NP adhesive disks were first sterilized and cleaned in an ultrasonic bath of 70 % ethanol for 30 min. The disks were then washed twice with sterile deionized water and air dried at room temperature.

#### Antibacterial treatment for comparison of viability assessment of planktonic bacteria with CFU counting, metabolic activity assays and Live/Dead staining

The bactericidal effect of photocatalytic treatment as a function of UV-A dose was evaluated with CFU counting, metabolic activity assays and Live/Dead staining combined with fluorescent intensity measurements. For antibacterial tests with both planktonic *S.* *epidermidis* and *S.* *mutans*, 10 μL of bacterial suspension (bacterial population ~10^7^) was spread on each NP adhesive disk using a pipette tip. The disks with bacteria were irradiated with a high-power UV-A diode (λ = 365 nm, NSCU033B(T), Nichia, Japan). A collimating lens ensured an even UV-A light intensity of 15 mW/cm^2^ over the irradiated area (UV light meter, UV-340, Lutron), and the treatment times were varied to provide UV-A doses ranging from 0 to 13.6 J/cm^2^. The 0 J/cm^2^ dose refers to control disks that were not exposed to UV-A light and were included to provide a reference level for determining the log reduction in viability of the samples subjected to UV-A irradiation. Four disks at each UV-A dose and for each bacteria strain were irradiated. The disks were inspected for moisture loss on the surface so that any bactericidal effect due to desiccation would be minimized. After photocatalytic treatment, each disk was immediately put into a well in a 48-well plate containing 100 μL of sterile water. The 48-well plate was then fixed to an incubating orbital shaker (Talboys, Troemner, USA) and shaken at 500 rpm for 2 min to re-suspend the bacteria from the disk surfaces. The sample disks were removed from the wells and bacterial viability was subsequently analyzed.

From the 100 μL of bacteria suspension of *S.* *epidermidis* after each test, 10 μL was taken for CFU counting, 10 μL for the metabolic assay incorporating resazurin and 50 μL for fluorescence intensity measurements following Live/Dead staining. From the 100 μL of bacteria suspension of *S.* *mutans* after each test, 10 μL was taken for the metabolic assay incorporating resazurin, 10 μL for the metabolic assay incorporating phenol red and 50 μL for fluorescence intensity measurements following Live/Dead staining.

#### Antibacterial treatment for comparison of viability assessment of planktonic bacteria with CFU counting and flow cytometry

To further assess Live/Dead staining, a comparison between CFU counting and flow cytometry was performed. Ten microliters of planktonic *S.* *epidermidis* bacterial suspension with a bacterial population of 10^8^ was spread on an NP adhesive disk using a pipette tip and illuminated with a UV-A dose of 42 J/cm^2^ to ensure a strong bactericidal effect. After the photocatalytic treatment, bacteria were re-suspended from the disk surface into 2 mL of sterile water. Ten microliters of bacterial suspension was taken for CFU counting, while the remainder was taken for Live/Dead staining and subsequent analysis with flow cytometry. To provide a control sample, a suspension of untreated planktonic *S.* *epidermidis* (10^8^ CFU in 2 mL) was analyzed with flow cytometry after Live/Dead staining.

#### Antibacterial treatment for assessing viability of an *S.* *mutans* biofilm with laser scanning confocal microscopy (LSCM)

The viability of 16-h-old *S.* *mutans* biofilm was assessed after photocatalytic treatment using LSCM. Three NP adhesive disks were first incubated with *S.* *mutans* (10^6^ CFU/mL) in BHIS broth for 4 h at 37 °C. The disks were then cultured in fresh BHIS broth in an orbital shaking incubator (100 rpm, Talboy) at 37 °C for 16 h to induce biofilm formation. For photocatalytic treatment, a UV-A irradiation dose of 40 J/cm^2^ was applied to one of the NP adhesive disks coated with the 16-h-old biofilm. The other biofilm-coated NP adhesive disks were used as a live control and a dead control in which the bacteria were killed by immersing in 70 % ethanol.

### Methods for analyzing bacterial viability after photocatalytic treatment

Six methods were used for assessing bacterial viability after photocatalytic treatment: CFU counting, metabolic activity assays based on resazurin and phenol red and Live/Dead staining viability assays combined with fluorescent intensity measurements, flow cytometry and LSCM.

#### CFU counting

Ten microliters of *S.* *epidermidis* bacterial suspension was taken from the bacterial suspension after the photocatalytic treatment for CFU counting. A dilution series was performed to achieve a suitable amount of bacteria on the LB agar plates (Sigma-Aldrich, Steinheim, Germany) for counting. The LB agar plates were cultured at 37 °C overnight and the resulting CFUs on the agar plates were imaged with a digital microscope (Dino Lite, Netherlands) and counted with the aid of the software Dotcount (developed by Martin Reuter, MIT, MA, USA). CFU counting was not used with *S.* *mutans* as testing showed a significant tendency for *S.* *mutans* cells to aggregate and form clumps of several cells, resulting in a gross underestimation of viability when using CFU counting.

#### Metabolic activity assay based on resazurin

The metabolic activity assay based on resazurin is an indirect method used to evaluate the viability of bacteria by measuring the accumulation of resorufin (pink in color and highly fluorescent), which is the reaction product of resazurin (blue in color and non-fluorescent) and reductive metabolic intermediates. Ten microliters of bacterial suspension was taken from the 100 μL bacterial suspension after the photocatalytic treatment for resazurin bacterial viability testing. For *S.* *epidermidis*, the 10 μL of bacterial suspension was added to 200 μL of MH broth containing resazurin (1.25 μg/mL) in a 96-well plate. A tenfold dilution series of living *S.* *epidermidis* bacterial suspension, from 10^7^ to 10 CFUs/well, was also prepared and placed in the 96-well plate to calibrate the number of surviving bacteria after photocatalytic treatment on the sample disks. The 96-well plate containing the resazurin assay was incubated at 37 °C, and color change (from blue to pink) due to reduction of non-fluorescent resazurin to pink resorufin by the bacterial metabolic activity was automatically recorded with a digital camera every 10 min. The initial number of surviving bacteria in the test wells was determined by comparing the time for color change to the *S.* *epidermidis* calibration series. The viability of *S.* *mutans* was evaluated with the same procedure used with *S.* *epidermidis* except that the assay culture media was BHI broth with 2.5 μg/mL resazurin instead of MH broth with 1.25 μg/mL resazurin.

#### Metabolic activity assay based on phenol red

The metabolic activity assay based on phenol red is an indirect method used to evaluate the amount of viable bacteria, which is related to a pH change in a culture medium containing the bacteria resulting from the accumulation of metabolic acid products. In this study, the phenol red assay was only used to determine the viability of *S.* *mutans* since they readily produce acidic metabolites. The assay changes color from red to yellow, due to accumulation of lactic acid, which is a sucrose metabolic by-product produced by *S.* *mutans*. The assay culture media consisted of BHI broth plus 2 % sucrose and 25 mg/L of the pH indicator phenol red (BHIS–PR broth). The pH of the BHIS–PR broth was adjusted to 7.10 before autoclaving. The same batch of BHIS–PR broth was used for both the calibration curve and viability testing to avoid variances caused by difference in broth media (All chemicals were obtained from Sigma-Aldrich, Steinheim, Germany).

Ten microliters of bacterial suspension was taken from 100 μL of bacterial suspension after photocatalytic treatment and added to 1.5 mL of BHIS–PR broth in a 48-well plate (Nunclon^®^ Δ Multidishes, Thermo Fisher Scientific, Germany). A calibration concentration series of *S.* *mutans* ranging from 10^7^ to 10 CFUs/well was also cultured parallel with the photocatalytic samples. The 48-well plate was incubated at 37 °C and the color of the wells containing the culture medium was automatically recorded every 10 min with a digital camera. The initial number of surviving bacteria in the test wells was determined by comparing the time of color change to the *S.* *mutans* calibration series.

#### Live/Dead staining combined with fluorescence intensity measurements

Fifty microliters of bacterial suspension was taken from the 100 μL bacterial suspension after photocatalytic treatment for assessment of viability with Live/Dead staining (Live/Dead^®^ *Bac*Light™ bacterial viability assay kit, L13152, Invitrogen, Eugene, USA). For both *S.* *epidermidis* and *S.* *mutans*, calibration curves were performed according to the product instructions. For each sample, fluorescence intensity was measured at an emission wavelength at 530 nm (green) and 620 nm (red) using an excitation wavelength at 485 nm (Infinite 200 microplate reader, Tecan, Switzerland). The ratio of green/red fluorescence intensities was calculated and compared to the calibration curves from samples of known viability to determine the viability of the photocatalytically treated bacteria.

#### Live/Dead staining combined with flow cytometery

After the photocatalytic treatment, approximately 2 mL of both untreated and treated *S.* *epidermidis* suspension was stained with Live/Dead stain according to the product instructions and the viability of the sample was determined with multi-laser analytical flow cytometry (LSR II, BD Biosciences).

#### Live/Dead staining combined with LSCM

After photocatalytic treatment, the *S.* *mutans* biofilm on the surface of the NP adhesive was stained with the Live/Dead staining kit according to the product instructions. A control NP adhesive disk with live 16-h-old *S.* *mutans* biofilm and a control NP adhesive disk with 16-h-old *S.* *mutans* biofilm treated with 70 % ethanol to kill the biofilm were also stained with the Live/Dead staining kit. The viability of the biofilm samples was assessed by imaging the samples with an LSCM (LSM 510 META, Carl Zeiss MicroImaging GmbH, Jena, Germany) using an excitation wavelength of 488 nm.

### Statistical tests

The Student’s *t* test was employed to determine if statistically significant differences existed between measured bactericidal effects using the different viability assessment methods.

## Results

### Viability measurements of planktonic *S.* *epidermidis* as a function of UV-A dose

**Fig. 1:**
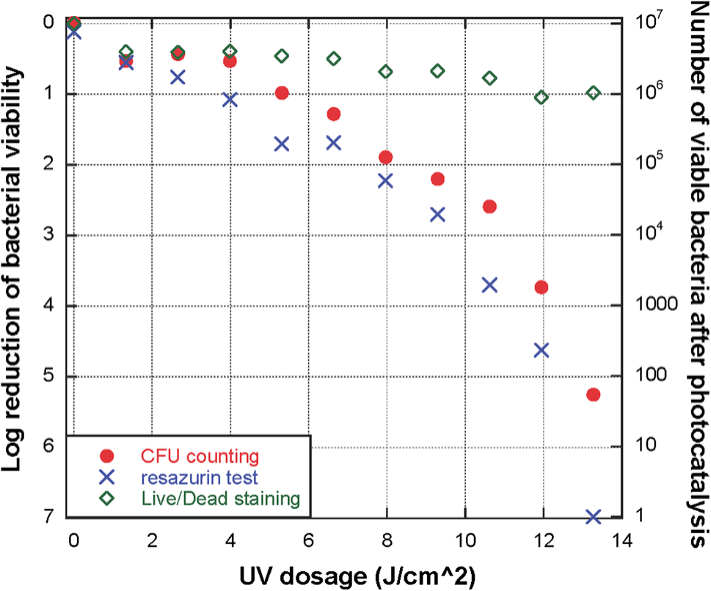
Bacterial viability of planktonic *S.* *epidermidis* after photocatalytic antibacterial treatment, measured with CFU counting, metabolic activity assay incorporating resazurin and Live/Dead staining. Each data point is the average of four tests; the standard deviations are within 0.63 log

Figure 1 shows the bacterial viability of planktonic *S.* *epidermidis* after photocatalytic antibacterial treatment, as measured with CFU counting, metabolic activity assay incorporating resazurin and Live/Dead staining with fluorescent intensity measurements.

The resazurin metabolic assay and CFU counting show a similar trend in that an increasing dose of UV-A irradiation leads to a greater antibacterial effect. It can be observed from Fig. 1 that an *S.* *epidermidis* population of ~10^7^ CFUs on the Φ 8 mm NP adhesive disks can be disinfected by photocatalysis with UV-A dose of 13.6 J/cm^2^ since a reduction of greater than 5 log was achieved. However, Live/Dead staining provided a much higher measure of bacterial viability at higher UV doses (less than 1 log reduction at UV doses of 4–13.6 J/cm^2^). For UV doses greater than 5 J/cm^2^, Live/Dead staining showed a statistically higher viability than both CFU counting and the resazurin assay (Student’s *t* test, *p* < 0.005).

### Viability measurements of planktonic *S.* *mutans* as a function of UV-A dose

**Fig. 2:**
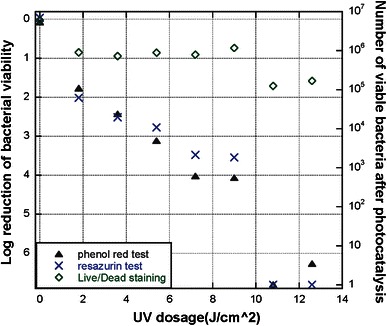
Bacterial viability of planktonic *S.* *mutans* after the photocatalytic antibacterial treatment, measured with metabolic activity assays incorporating phenol red and resazurin, respectively, and Live/Dead staining. Each data point is the average of four tests; the standard deviations are within 0.88 log

Figure 2 shows the quantification of viable planktonic *S.* *mutans* after photocatalytic treatment with UV-A doses ranging from 0 to 13.6 J/cm^2^. For assessing *S.* *mutans* viability, three methods were employed: metabolic activity assay based on phenol red, metabolic activity assay based on resazurin and Live/Dead staining.

The metabolic assays incorporating phenol red and resazurin provided similar measures of bacterial viability, which indicate that an increasing dose of UV-A irradiation leads to an increasing antibacterial effect, as expected. From Fig. 2, it can be observed that an *S.* *mutans* population of ~10^7^ CFUs on Φ 8 mm NP adhesive disks can be disinfected (>5 log reduction) by photocatalysis with a UV-A dose of 10 J/cm^2^ or greater. As with the viability measurements of *S.* *epidermidis*, Live/Dead staining indicates a much higher bacterial viability than the metabolic assays, see Fig. 2. For all tested non-zero UV doses, Live/Dead staining showed a statistically higher viability than both the resazurin and phenol red assays (Student’s *t* test, *p* < 0.005). With a UV-A dose of 13.6 J/cm^2^, Live/Dead staining shows less than a 2 log reduction of bacteria, while both metabolic activity assays show more than 6 log bacterial reduction.

### Bacterial viability evaluation of photocatalytically treated planktonic *S.* *epidermidis* based on Live/Dead staining and flow cytometry

**Fig. 3:**
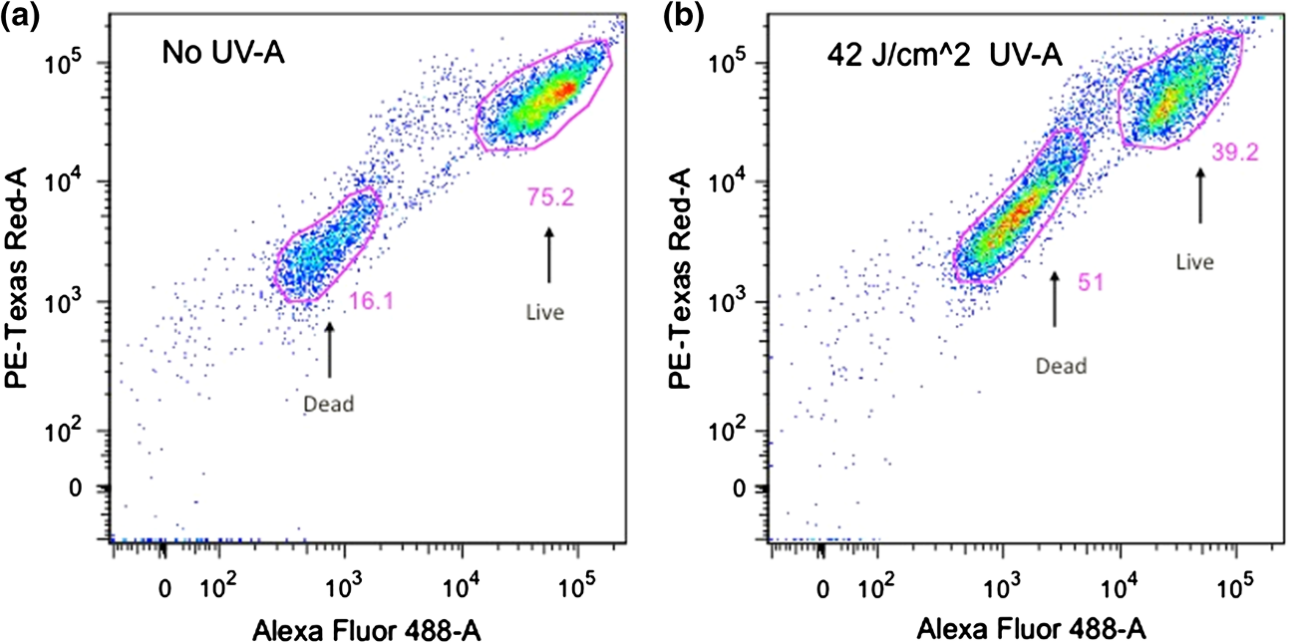
Planktonic *S.* *epidermidis* viability assessed with flow cytometry utilizing Live/Dead staining. **a** A control sample of *S.* *epidermidis* without photocatalytic treatment. **b** The viability of an *S.* *epidermidis* sample subjected to a UV-A irradiation dose of 42 J/cm^2^ on an NP adhesive disk

Figure 3 shows flow cytometry analysis of a control *S.* *epidermidis* sample (a) and an *S.* *epidermidis* sample after being subject to a UV-A dose of 42 J/cm^2^ (b). Both samples were stained with the Live/Dead stain kit prior to analysis with flow cytometry. In Fig. 3a, in which the *S.* *epidermidis* sample was not treated with UV-A light, it could be observed that 16 % of the cells were non-viable while 75 % were active. Figure 3b shows that after a UV-A irradiation dose of 42 J/cm^2^, 51 % of *S.* *epidermidis* population was non-viable while 39 % was alive. However, a CFU counting analysis of the same sample of photocatalytically treated *S. epidermidis* displayed in Fig. 3b showed that only 7 of 10^6^ bacteria survived. The results from CFU counting are in contrast to the Live/Dead staining results when combined with flow cytometry as shown in Fig. 3b.

### Bacterial viability evaluation of photocatalytically treated *S.* *mutans* biofilm based on Live/Dead staining and LSCM

**Fig. 4:**
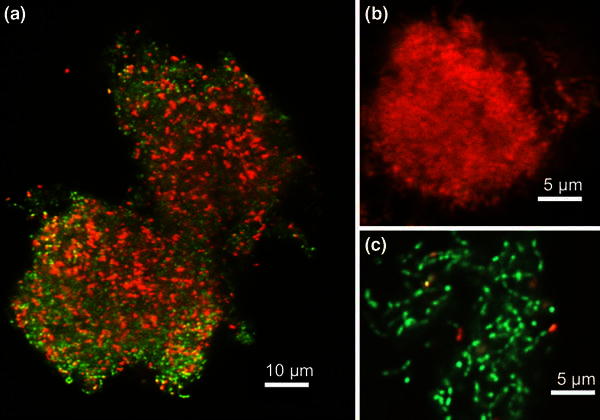
*S.* *mutans* biofilm with Live/Dead staining and imaged with LSCM. The *green* signal is due to the dye SYTO9, indicating alive cells while the *red* signal is due to propidium iodide which marks the dead cells. **a** Photocatalytically treated biofilm with a UV-A irradiation dose of 40 J/cm^2^; **b** control of dead biofilm; **c** control of living biofilm

Figure 4a shows an LSCM image of a photocatalytically treated *S.* *mutans* biofilm after a UV-A irradiation dose of 40 J/cm^2^. Figure 4b shows an LSCM image from an *S.* *mutans* biofilm treated with 70 % ethanol and Fig. 4c displays an LSCM image of an untreated *S.* *mutans* biofilm. From the figures, green and/or red signals can be observed, which represent living and dead bacteria, respectively. Thus, Fig. 4b indicates that the 70 % ethanol treatment effectively killed the biofilm since no green signal was observed. Conversely, Fig. 4a shows qualitatively that a large part of the photocatalytically treated biofilm is alive.

## Discussion

In this work, different methods were used in the analysis of *S.* *epidermidis* and *S.* *mutans* bacterial viability after photocatalysis treatment. An important issue raised from the above results is the disagreement of Live/Dead staining data compared to both CFU counting and the two types of metabolic activity assays. CFU counting, the resazurin assay and the phenol red assay all showed the same tendency of bacterial viability to decrease with a corresponding increase in UV-A light irradiation, whereas Live/Dead staining indicated a much higher level of viability in the bacteria samples subjected to photocatalytic treatment. This tendency for Live/Dead staining to indicate a higher viability in photocatalytically treated bacteria compared to other methods can be observed in Figs. 1 and 2 where Live/Dead staining was quantified through fluorescent intensity measurements in a multiplate reader and in Fig. 3 where Live/Dead staining was combined with flow cytometry. From Fig. 4, it can even be seen that LSCM with Live/Dead staining showed a high degree of viability in an *S.* *mutans* biofilm that had been subjected to photocatalytic treatment with a high UV-A dose. It has been previously shown that a similar UV-A dose on *S.* *mutans* biofilm cultured on NP adhesives has a potent bactericidal effect (Cai et al. 2013). In these tests, a metabolic activity assay incorporating phenol red was used to assess viability and showed a 5 log reduction in viability.

The reason for the discrepancy between the Live/Dead staining results and other methods could be related to the criteria for bacterial viability utilized by the different methods. For example, CFU counting examines the number of viable bacteria that can form colonies on a broth agar plate, while metabolic activity assays assess the accumulation of metabolic product or intermediate, which depends on both the number and metabolic rate of bacteria. As mentioned previously. CFU counting can sometimes provide an underestimation of viability if the bacterial cells aggregate, and this was the reason *S.* *mutans* was not assessed with CFU counting. On the other hand, when comparing the CFU results with the resazurin assay for planktonic *S.* *epidermidis*, similar estimations of viability were found, where the CFU counting results indicated slightly higher viability. Live/Dead staining is based on assessment of the bacterial membrane integrity with the help of two nucleic acid dyes, SYTO 9 and propidium iodide. SYTO9 can permeate the cell membrane of both dead and living cells, while propidium iodide can only permeate damaged cell membranes, resulting in dead bacteria producing a red fluorescence signal and live bacteria producing a green signal. It is generally accepted that ROS generated during TiO_2_ photocatalysis attack the bacterial cell wall and/or membrane and are responsible for killing the bacteria (Maness et al. 1999). However, it appears that even though the ROS attack causes a reduction or total loss of normal cellular function, the membrane integrity (as probed by propidium iodide) may not be significantly affected. Regardless of the mechanism that gives rise to the higher measure of viability when using Live/Dead staining, the results in this study suggest that this method may not be suitable for the analysis of bacterial viability following photocatalytic treatments.

Returning to Fig. 1, it is interesting to note that viability assessed with the resazurin assay consistently showed a slightly lower viability than that determined from CFU counting, for tests involving UV-A irradiation. A possible explanation for this could be related to the recovery of some bacteria subjected to the photocatalytic treatment. It is known that bacteria that are sub-lethally injured due to ROS exposure can recover under optimum environmental conditions (Rizzo 2009). This would result in a delay of growth and division and consequently exhibit itself as a lower signal in a metabolic assay due to the lower/delayed metabolic activity of the affected cells. However, CFU counting would not necessarily distinguish between a healthy cell and a damaged cell that recovered from its injuries if both result in a countable colony at a later time point. Support for this hypothesis was found by observing the agar plates containing the *S.* *epidermidis* samples during the incubation time prior to CFU counting. While colonies formed by a control sample of healthy *S.* *epidermidis* not subjected to UV-A light appeared on the agar plate at approximately the same time and were of the same size at the time of counting, colonies formed from samples subjected to UV-A irradiation appeared visibly at different times during the incubation period and were of different sizes at the time of counting.

When choosing an appropriate method for assessing viability in antibacterial testing, it is important to consider the mechanism by which the method probes viability. Often a combination of methods is required to give a more certain indication of viability. Each method has unique criteria for determining bacterial viability. CFU counting shows the number of living bacteria; metabolic activity assays show the multiplication and metabolic rate of an amount of living bacteria; and molecular probe methods examine the membrane integrity. The sensitivity of the various methods is also an important issue to consider in practice. For example, CFU counting is suitable for examining very low concentrations of living bacteria, but is only reliable for assessing bacterial populations where one can be certain that individual cells can be well separated from each other on the plate. Metabolic activity assays are also applicable for sample showing more than 6 log reduction of viability, as demonstrated in this study, and because the technique can largely avoid sample manipulation (Pantanella et al. 2008), it is suitable for assessing the viability of both planktonic and biofilm forms of bacteria. For the antibacterial tests not involving photocatalysis, Live/Dead staining has been used to analyze viability, visualize both viability and distribution of live and dead cells and analyze samples containing multiple bacterial species.

## Conclusions

Multiple methods were compared for the assessment of bacterial viability after photocatalytic treatment. The results of CFU counting and metabolic activity assays incorporating resazurin and phenol red showed good agreement with each other, while tests based on the Live/Dead staining differed significantly, showing a much higher viability. Our results suggest that the use of Live/Dead staining may not be applicable to the assessment of bacterial viability following antibacterial photocatalytic treatments. The present findings are expected to become valuable for the development and evaluation of photocatalytically based sterilization applications in, e.g., medicine and dentistry.

## References

[CR1] Allahverdiyev AM, Abamor ES, Bagirova M, Rafailovich M (2011). Antimicrobial effects of TiO_2_ and Ag_2_O nanoparticles against drug-resistant bacteria and leishmania parasites. Future Microbiol.

[CR2] Asadishad B, Ghoshal S, Tufenkji N (2011). Method for the direct observation and quantification of survival of bacteria attached to negatively or positively charged surfaces in an aqueous medium. Environ Sci Technol.

[CR3] Banas JA (2004). Virulence properties of *Streptococcus mutans*. Front Biosci.

[CR4] Bar W, Bade-Schumann U, Krebs A, Cromme L (2009). Rapid method for detection of minimal bactericidal concentration of antibiotics. J Microbiol Methods.

[CR5] Belanger PA, Beaudin J, Roy S (2011). High-throughput screening of microbial adaptation to environmental stress. J Microbiol Methods.

[CR6] Berney M, Hammes F, Bosshard F, Weilenmann HU, Egli T (2007). Assessment and interpretation of bacterial viability by using the LIVE/DEAD *Bac*Light kit in combination with flow cytometry. Appl Environ Microbiol.

[CR7] Bettencourt P, Pires D, Carmo N, Anes E (2010). Application of confocal microscopy for quantification of intracellular mycobacteria in macrophages.

[CR8] Blake DM, Maness PC, Huang Z, Wolfrum EJ, Huang J, Jacoby WA (1999). Application of the photocatalytic chemistry of titanium dioxide to disinfection and the killing of cancer cells. Sep Purif Method.

[CR9] Cai YL, Strømme M, Melhus Å, Engqvist H, Welch K (2013) Photocatalytic inactivation of biofilms on bioactive dental adhesives. J Biomed Mater Res B (in press) 10.1002/jbm.b.32980 23847027

[CR10] Chatterjee D, Dasgupta S (2005). Visible light induced photocatalytic degradation of organic pollutants. J Phototch Photobio C.

[CR11] Chen J, Poon CS (2009). Photocatalytic construction and building materials: from fundamentals to applications. Build Environ.

[CR12] Collinge CA, Goll G, Seligson D, Easley KJ (1994). Pin tract infections: silver vs uncoated pins. Orthopedics.

[CR13] Diaper JP, Tither K, Edwards C (1992). Rapid assessment of bacterial viability by flow-cytometry. Appl Microbiol Biotechnol.

[CR14] Donlan RM (2001). Biofilm formation: a clinically relevant microbiological process. Clin Infect Dis.

[CR15] Fujishima A, Zhang XT, Tryk DA (2008). TiO_2_ photocatalysis and related surface phenomena. Surf Sci Rep.

[CR16] Jie H, Lee HB, Chae KH, Huh MY, Matsuoka M, Cho SH, Park JK (2012). Nitrogen-doped TiO_2_ nanopowders prepared by chemical vapor synthesis: band structure and photocatalytic activity under visible light. Res Chem Intermediat.

[CR17] Kirchnerova J, Cohen MLH, Guy C, Klvana D (2005). Photocatalytic oxidation of *n*-butanol under fluorescent visible light lamp over commercial TiO_2_ (Hombicat UV100 and Degussa P25). Appl Catal A-Gen.

[CR18] Li QL, Mahendra S, Lyon DY, Brunet L, Liga MV, Li D, Alvarez PJJ (2008). Antimicrobial nanomaterials for water disinfection and microbial control: potential applications and implications. Water Res.

[CR19] Lilja M, Welch K, Astrand M, Engqvist H, Strømme M (2012). Effect of deposition parameters on the photocatalytic activity and bioactivity of TiO_2_ thin films deposited by vacuum arc on Ti-6Al-4V substrates. J Biomed Mater Res B.

[CR20] Lisle JT, Pyle BH, McFeters GA (1999). The use of multiple indices of physiological activity to access viability in chlorine disinfected *Escherichia coli* O157: H7. Lett Appl Microbiol.

[CR21] Mah TFC, O’Toole GA (2001). Mechanisms of biofilm resistance to antimicrobial agents. Trends Microbiol.

[CR22] Mahan J, Seligson D, Henry SL, Hynes P, Dobbins J (1991). Factors in pin tract infections. Orthopedics.

[CR23] Maness PC, Smolinski S, Blake DM, Huang Z, Wolfrum EJ, Jacoby WA (1999). Bactericidal activity of photocatalytic TiO_2_ reaction: toward an understanding of its killing mechanism. Appl Environ Microbiol.

[CR24] Nah YC, Paramasivam I, Schmuki P (2010). Doped TiO_2_ and TiO_2_ nanotubes: synthesis and applications. Chem Phys Chem.

[CR25] Ohno T, Sarukawa K, Tokieda K, Matsumura M (2001). Morphology of a TiO_2_ photocatalyst (Degussa, P-25) consisting of anatase and rutile crystalline phases. J Catal.

[CR26] Pantanella F, Valenti P, Frioni A, Natalizi T, Coltella L, Berlutti F (2008). BibTimer Assay, a new method for counting *Staphylococcus* spp. in biofilm without sample manipulation applied to evaluate antibiotic susceptibility of biofilm. J Microbiol Methods.

[CR27] Peeters E, Nelis HJ, Coenye T (2008). Comparison of multiple methods for quantification of microbial biofilms grown in microtiter plates. J Microbiol Methods.

[CR28] Rizzo L (2009). Inactivation and injury of total coliform bacteria after primary disinfection of drinking water by TiO_2_ photocatalysis. J Hazard Mater.

[CR29] Robertson PKJ, Robertson JMC, Bahnemann DW (2012). Removal of microorganisms and their chemical metabolites from water using semiconductor photocatalysis. J Hazard Mater.

[CR30] Sanchez B, Sanchez-Munoz M, Munoz-Vicente M, Cobas G, Portela R, Suarez S, Gonzalez AE, Rodriguez N, Amils R (2012). Photocatalytic elimination of indoor air biological and chemical pollution in realistic conditions. Chemosphere.

[CR31] Sandberg ME, Schellmann D, Brunhofer G, Erker T, Busygin I, Leino R, Vuorela PM, Fallarero A (2009). Pros and cons of using resazurin staining for quantification of viable *Staphylococcus aureus* biofilms in a screening assay. J Microbiol Methods.

[CR32] Sheng GD, Li JX, Wang SW, Wang XK (2009). Modification to promote visible-light catalytic activity of TiO_2_. Prog Chem.

[CR33] Welch K, Cai YL, Engqvist H, Strømme M (2010). Dental adhesives with bioactive and on-demand bactericidal properties. Dent Mater.

[CR34] Welch K, Cai YL, Strømme M (2012). A method for quantitative determination of biofilm viability. J Funct Biomater.

[CR35] Wierzchos J, De los Rios A, Sancho LG, Ascaso C (2004). Viability of endolithic micro-organisms in rocks from the McMurdo Dry Valleys of Antarctica established by confocal and fluorescence microscopy. J Microsc.

